# The Association Between Nutritional Risk and Bone Stiffness in Elderly Men and Women in a Population-Based Study in Northeast Germany

**DOI:** 10.3390/nu16244288

**Published:** 2024-12-12

**Authors:** Jannis Riest, Nele Friedrich, Matthias Nauck, Henry Völzke, Simone Gärtner, Anke Hannemann

**Affiliations:** 1 Institute of Clinical Chemistry and Laboratory Medicine, University Medicine Greifswald, 17475 Greifswald, Germany; 2 DZHK (German Centre for Cardiovascular Research), Partner Site Greifswald, University Medicine Greifswald, 17475 Greifswald, Germany; 3 Institute for Community Medicine, University Medicine Greifswald, 17475 Greifswald, Germany; 4 Department of Internal Medicine A, University Medicine Greifswald, 17475 Greifswald, Germany

**Keywords:** nutritional risk, quantitative ultrasound, bone stiffness, elderly men and women, osteoporosis, BMI, underweight

## Abstract

Background: The Geriatric Nutritional Risk Index (GNRI) has shown promising potential for identifying individuals at risk for osteoporosis in various patient cohorts. However, data from the general population confirming or refuting the usefulness of the GNRI as a risk factor for osteoporosis are sparse. We therefore aimed to clarify whether the GNRI is associated with the ultrasound-based bone stiffness index and the osteoporotic fracture risk in a sample of elderly men and women from the general population. Methods: Data from 1417 participants in the Study of Health in Pomerania START-2 or TREND-0 aged 65 years or older with quantitative ultrasound measurements at the heel and GNRI values were examined. In cross-sectional linear and logistic regression models, associations between the GNRI and heel stiffness index or ultrasound-based osteoporotic fracture risk were examined. All analyses were repeated after stratification of the study population according to BMI (underweight/normal weight, overweight and obese). Results: In underweight/normal weight individuals, higher, i.e., better, GNRI values had a positive effect on the stiffness index (β-coefficient per standard deviation increase in GNRI = 2.69, standard error = 1.00, *p* = 0.007). With increasing GNRI values, underweight/normal weight elderly men and women also had higher chances of a low osteoporotic fracture risk (odds ratio 1.42, 95% confidence interval 1.04–1.94, *p* = 0.026). Corresponding associations in overweight or obese individuals were absent. Conclusions: In elderly men and women with underweight/normal weight, the GNRI is positively associated with the bone stiffness index and the related osteoporotic fracture risk. In this group, the GNRI may prove useful in identifying individuals with an elevated fracture risk.

## 1. Introduction

In Germany and worldwide, osteoporosis is a growing public health concern. An estimated 5.6 million men and women over the age of 50 years suffered from osteoporosis in Germany in 2019 [1], accounting for 4% (13.8 billion €) of German healthcare costs in that year [2]. The consequences of osteoporotic fractures for affected patients are profound: pain, deterioration of quality of life and increased mortality [3], particularly in patients with hip fractures [4].

Osteoporosis is diagnosed using the dual-energy X-ray absorptiometry (DXA) T-score in concert with clinical and anamnestic findings [5]. Numerous risk factors contribute to the development of osteoporosis, including age, female sex, low body mass index (BMI), history of fragility fractures, smoking, excessive alcohol consumption, positive family history of osteoporosis and glucocorticoid treatment [5]. Regarding nutrition, calcium, vitamin D and protein intake play an important role in maintaining bone health in younger as well as older people. Indeed, in prevention and treatment for individuals with high osteoporotic fracture risk, nutritional intake or supplementation plays an important role complementary to that of pharmacotherapy [6].

In order to assess nutrition-related risk in elderly hospitalized patients, Bouillanne et al. created the Geriatric Nutritional Risk Index (GNRI) [7]. It is based on the nutritional risk index by Buzby et al. [8] but uses ideal weight instead of usual weight. The GNRI is an economic, efficient and safe tool to predict nutrition-related morbidity and mortality in elderly hospitalized patients [7]. Meanwhile, it has been associated not only with cancer outcomes [9], depression [10] and cardiovascular events [11] but also with the presence of osteoporosis in type 2 diabetes [12]. Furthermore, the GNRI was shown to possess a higher predictive value for osteoporosis than two other nutritional risk assessment tools [12]. Above this, it has been associated with bone mineral density (BMD) in various patient cohorts, including patients undergoing haemodialysis [13,14,15,16] or total thyroidectomy [17], patients with type 2 diabetes [12,18,19,20] and patients with rheumatoid arthritis on disease-modifying anti-rheumatic drugs [21].

In ageing populations, the prevalence of osteoporosis and osteoporotic fractures rises. Projections suggest an increase in the annual number of osteoporotic fractures in the EU27+2 from 4.28 million in 2019 to 5.05 million in 2034 [1]. In light of the high individual and societal costs, it is of paramount importance to prioritise the prevention of osteoporotic fractures. This, in turn, requires identifying patients at risk. As stated above, low body weight and poor nutritional quality are among the modifiable risk factors for osteoporosis [5]. Overweight is, to a certain degree, protective for bone health due to the positive relation between BMI and BMD [22]. Yet, BMI alone is not a reliable predictor of osteoporotic fractures, as the association between body anthropometry and fracture risk is complex [23]. Adding information on nutritional deficiencies may allow a better evaluation of individual risk, and the GNRI may offer such a possibility. Yet, the associations between the GNRI and BMD or osteoporosis have rarely been examined in the general population [24,25,26]. In one of these rare studies, Wang et al. found a positive association between GNRI and BMD in multiple femoral locations, as well as an inverse association with the presence of osteoporosis in postmenopausal women [26]. In another study, Qing et al. revealed a positive correlation between GNRI values and DXA-based T-scores at all analysed anatomical sites except for L4 in elderly Chinese people [25]. Studies analysing the relation between the GNRI and bone stiffness assessed by quantitative ultrasound (QUS), which allows fracture risk prediction without ionising radiation, do not exist to our knowledge. To evaluate the relationship between GNRI and QUS measurements and clarify the association of GNRI and bone parameters in the general population, we analysed the associations between the GNRI and the QUS-based stiffness index as well as the osteoporotic fracture risk in elderly participants from two population-based cohorts in northeast Germany.

## 2. Materials and Methods

### 2.1. The Study of Health in Pomerania (SHIP)

SHIP is a population-based project in Northeast Germany, including adult participants [27]. For the present analyses, data from the SHIP-START and the SHIP-TREND cohorts were obtained. The study population of both cohorts was selected based on representative samples of the 20- to 79-year-old inhabitants of the study region, including the cities of Greifswald, Stralsund and Anklam and the surrounding communities. From these samples, 4308 (SHIP-START) and 4420 (SHIP-TREND) individuals participated in the baseline examinations in 1997–2001 (SHIP-START-0) and in 2008–2012 (SHIP-TREND-0). QUS measurements were restricted to the second follow-up of the SHIP-START cohort (SHIP-START-2, n = 2333) and the baseline examination of the SHIP-TREND cohort. These examinations were used in the following study.

Written informed consent was obtained from all individual participants included in the study. All procedures performed in studies involving human participants were in accordance with the ethical standards of the institutional and/or national research committee and with the 1964 Declaration of Helsinki and its later amendments or comparable ethical standards. Further details on the study design, sampling procedures and rationale are given elsewhere [27].

### 2.2. Study Population

SHIP offers a broad range of medical examinations to the study participants. This includes, for example, the collection of biological samples; instruments to assess somatometric measurements and cardiovascular health and a computer-assisted personal interview on sociodemographics, medical history and lifestyle.

A total of N = 6753 adult men and women participated in SHIP-START-2 and SHIP-TREND-0. Among these, n = 4875 were younger than 65 years and therefore excluded from the present analyses. From the remaining n = 1878 individuals aged 65–90 years or older, we further excluded all participants who reported intake of medication that alters bone metabolism, i.e., glucocorticoids for systemic use or anti-osteoporotic drugs (n = 92), as well as those who had missing data on the GNRI (n = 60), QUS measurements (n = 268) or any of the confounders described below (n = 40). After additionally excluding one participant with extremely high BMI (>50 kg/m^2^), the final study population comprised 1417 individuals.

### 2.3. Measurements

Participants were classified as physically inactive when they reported less than one hour of regular physical activity per week during summer and winter. Intake of medication was recorded and classified using the anatomical therapeutic chemical classification system (ATC). Glucocorticoids for systemic use were defined as ATC codes H02AB and H02BX. Anti-osteoporotic drugs were defined as bisphosphonates (ATC codes M05BA and M05BB), selective oestrogen receptor modulators (ATC code G03XC) or parathyroid hormone and analogues (ATC code H05AA). Menopausal hormone therapy was defined as ATC codes G03C, G03D and G03F. Diabetes mellitus was defined as being present when participants reported a respective physician’s diagnosis, reported intake of antidiabetic medication (ATC code A10), had HbA1c ≥ 6.5% or had a random glucose concentration ≥11.1 mmol/L. Liver disease was defined as being present when participants reported a diagnosis of liver cirrhosis, hepatitis or fatty liver disease. Standardized measurements of body height and weight were performed with calibrated scales. BMI was calculated as weight (kg)/height^2^ (m^2^). Underweight/normal weight was defined as BMI < 25 kg/m^2^, overweight as BMI ≥ 25 to <30 kg/m^2^ and obesity as BMI ≥ 30 kg/m^2^.

Venous blood samples were taken from the cubital vein of participants in the supine position. Blood sampling was performed in the mornings. Serum albumin and creatinine concentrations were measured on the Dimension Vista 1500 system (Siemens Healthineers, Erlangen, Germany). Serum creatinine was measured enzymatically, and the estimated glomerular filtration rate (eGFR) was calculated according to the CKD-EPI formula [28]. The GNRI was calculated according to the following formula [7]: GNRI = (1.489 × albumin [g/L]) + (41.7 × (weight [kg]/ideal weight [kg]), with ideal weight calculated according to the Lorentz formula [29]. For men, ideal weight is calculated as (height [cm] − 100) − ((height [cm] − 150)/4); for women, it is calculated as (height [cm] − 100) − ((height [cm] − 150)/2). In accordance with Bouillanne et al. [7], the ratio of actual weight to ideal weight was set to ‘1’ in individuals who exceeded their ideal weight. GNRI values ranged between 83.4 and 113.2 (mean: 99.5, SD 4.2) and were used to define the individual nutrition-related risk according to the following categories: no risk (GNRI ≥ 98), low risk (GNRI 92 to <98), moderate risk (GNRI 82 to <92) and high risk (GNRI < 82).

### 2.4. QUS Measurements

BMD measurements using DXA represent the gold standard in the diagnosis and monitoring of osteoporosis. However, DXA measurements utilize low doses of ionizing radiation. The exposure of our participants to this procedure without medical necessity gave rise to ethical concerns. In light of the numerous advantages of QUS in population-based settings, including its good predictive value for osteoporotic fractures [30], its ease of use, its short testing time and its cost-effectiveness, we chose to use QUS for the evaluation of bone health in our participants. The QUS measurements were performed using the Achilles InSight (GE Medical Systems Ultrasound, GE Healthcare, Chalfont St. Giles, UK), as previously described [31]. In short, the Achilles InSight is a water-based bone ultrasonometer that measures the speed of sound and the frequency-dependent attenuation of the sound waves that pass through an individual’s heel (os calcis). The two characteristics are combined to form the stiffness index according to the following formula: stiffness index = (0.67 × broadband ultrasound attenuation) + (0.28 × speed of sound) − 420. Individual stiffness index values are then compared to values obtained in a healthy young reference population. Indices of −2.5 standard deviations (SD) compared to the reference mean indicate a high osteoporotic fracture risk, indices of −1 to −2.5 SD indicate a medium osteoporotic fracture risk and indices above −1 SD indicate a low osteoporotic fracture risk. QUS measurements were performed successively on both feet by trained and certified examiners. The data from the foot with the lower stiffness index were used for statistical analyses.

### 2.5. Statistical Analysis

General characteristics of the study population according to GNRI categories are presented as the mean and SD or proportions. Group differences were tested with *t*-tests (continuous data) or chi-squared tests (categorical data). *p*-values < 0.05 were considered statistically significant.

In linear regression models, cross-sectional associations between the GNRI (exposure) and the ultrasound-based stiffness index (outcome) were assessed. Subsequently, in cross-sectional logistic regression models, associations between the GNRI and the ultrasound-based osteoporotic fracture risk were assessed. From the linear regression models, β coefficients with standard errors are reported, and from the logistic models, odds ratios (ORs) with 95% confidence intervals (CIs) are reported. In all models, the effects of a one-SD increase in the GNRI, which equals 4.24 points, were estimated. For the logistic models, the ultrasound-based osteoporotic fracture risk was dichotomized into low and moderate/high risk. Moderate/high risk was set as the reference category.

All regression models were adjusted for sex (male/female), age (in years), physical activity (active/inactive), diabetes mellitus (yes/no), liver disease (yes/no), eGFR (in mL/min/1.73 m^2^), menopausal hormone therapy (yes/no) and BMI (in kg/m^2^). We further investigated whether effect modification by any of the covariates was present in the linear regression models. Interaction terms were not significant (*p* > 0.05) for any of the covariates except BMI, when it was entered in the models as a categorical variable (*p* = 0.03). All models were therefore calculated in the whole population and in subgroups according to BMI categories. All statistical analyses were performed with SAS 9.4 (SAS Institute Inc., Cary, NC, USA).

## 3. Results

### 3.1. Characteristics of the Study Population

The study population included 755 men and 662 women, aged 65 to 90 years. Two out of three participants (n = 955; 67.3%) had a GNRI ≥ 98 and no nutritional risk, about 30% (n = 422) had a GNRI between 92 and <98 and a low nutritional risk and about 3% (n = 40) had a GNRI between 82 and <92 and a moderate nutritional risk. None of the participants had a high nutritional risk. Due to the low number of participants with a moderate nutritional risk, the categories of low and moderate nutritional risk were pooled.

Table 1 shows the comparison of participants without and those with low/moderate nutritional risk. While the sex-distribution was similar between both groups, participants with low/moderate risk were slightly older, had a higher BMI, were more often physically inactive, had more often diabetes mellitus, were more often smokers and had a lower eGFR than participants without nutritional risk. Differences in the ultrasound-based stiffness index or fracture risk were, however, not statistically significant between the two groups.

### 3.2. GNRI and QUS-Based Stiffness Index

In the fully adjusted linear regression model, an association between the GNRI and the ultrasound-based stiffness index was absent. Yet, in the subgroup analyses assessing individuals with underweight/normal weight (n = 240), overweight (n = 653) and obesity (n = 524) separately, a statistically significant positive association was found.

**Figure 1:**
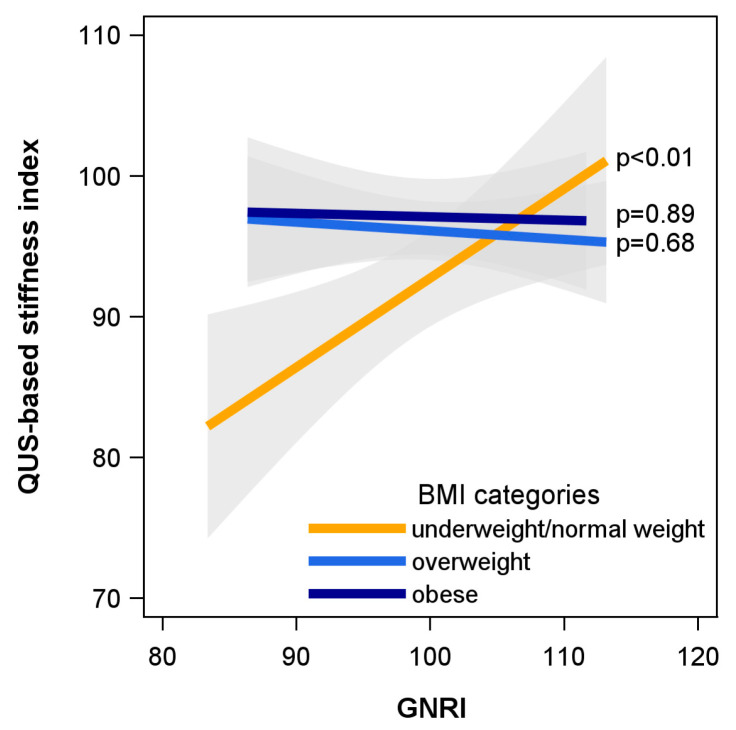
Associations between the GNRI and the ultrasound-based stiffness index in underweight/normal weight (n = 240), overweight (n = 653) or obese (n = 524) study participants. Illustrated is the estimated stiffness index (solid line) with 95% confidence intervals (shaded area) for an average male study participant (aged 71 years, physically active, without diabetes mellitus or liver disease, with an eGFR of 71 mL/min/1.73 m^2^) with a BMI of 23, 28 or 33 kg/m^2^. BMI, body mass index; eGFR, estimated glomerular filtration rate; GNRI, Geriatric Nutritional Risk Index; QUS, quantitative ultrasound.

In underweight/normal weight participants, an increase of 1 SD (4.24 points) in the GNRI was associated with an increase of 2.69 points in the stiffness index (standard error = 1.00, *p* = 0.007). Thus, in these individuals, a better GNRI was related to higher bone stiffness, while a worse GNRI was related to lower bone stiffness. An increase in BMI, however, was unrelated to bone stiffness (β per SD increase = 1.79, std err = 2.85, *p* = 0.531) in underweight/normal weight participants. In overweight and obese individuals, no associations between GNRI and bone stiffness were found (Table 2, Figure 1).

### 3.3. GNRI and QUS-Based Osteoporotic Fracture Risk

About half of the study population had a low ultrasound-based osteoporotic fracture risk (49.0%), about 40% had a moderate risk and about 11% had a high risk. In the logistic regression model for the whole population, the GNRI was not associated with the ultrasound-based fracture risk (Table 2). Again, however, subgroup analyses according to BMI revealed that in the underweight/normal weight participants, an increase in the GNRI was related to higher chances for a low fracture risk (OR 1.42, 95% CI 1.04–1.94). Similarly, it was related to lower chances for a moderate/high fracture risk (OR 0.70, 95% CI 0.52–0.96). While this association was on the threshold of statistical significance (*p* = 0.026), it fits well with the above findings from the linear model. In overweight and obese participants, associations were absent.

**Figure 2:**
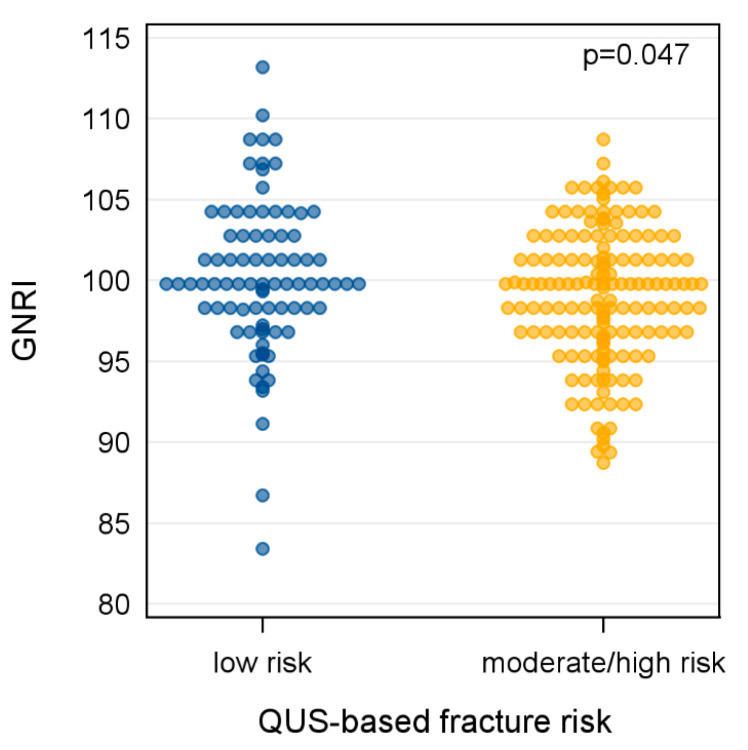
Jitter plot illustrating the distribution of the GNRI according to the ultrasound-based osteoporotic fracture risk in 240 underweight/normal weight study participants. A *t*-test was used to compare group differences. GNRI, Geriatric Nutritional Risk Index; QUS, quantitative ultrasound.

Finally, we closely examined the group of underweight/normal weight participants. Figure 2 displays the distribution of GNRI according to the ultrasound-based fracture risk in these individuals. Among the 240 individuals, 84 had a low fracture risk and 156 had a moderate/high fracture risk. The average GNRI was higher in participants with a low fracture risk (mean GNRI = 100.2, SD = 4.8) than in participants with a moderate/high fracture risk (mean GNRI = 99.0, SD = 4.1). A *t*-test confirmed the statistical significance of the group differences (*p* < 0.05).

## 4. Discussion

Our study, examining elderly men and women from the general population, revealed a potentially protective effect of a higher GNRI on the ultrasound-based bone stiffness index. This effect was, however, restricted to a small number of underweight/normal weight individuals, while the GNRI had no effect in overweight or obese individuals.

The association of the GNRI with BMD has been investigated in various populations. In small studies examining patients undergoing haemodialysis or total thyroidectomy or having rheumatoid arthritis, a low GNRI was associated with lower BMD or T-score in the femoral neck [13,14,15,21], lumbar spine [13,14,15,17], distal radius [13,15] or total hip [13] and with a higher prevalence of osteopenia [14] and osteoporosis [13,14]. Additionally, in patients with type 2 diabetes, results were quite consistent: the GNRI was found to be associated with BMD at all considered sites in several studies, e.g., [18,19,20], and with the development of osteoporosis [12,18,19]. Only one previous study, including haemodialysis patients, reported no association of GNRI with BMD in the lumbar spine or femoral neck [16].

Despite these promising previous results, only a few studies have evaluated the relation of GNRI and bone health in the general population. This includes one study using Chinese data [25] and two studies using US data [24,26]. In 1130 elderly Chinese men and women (60–89 years), Qing et al. [25] found stable positive associations between GNRI and BMD in the total hip in both sexes, while associations with BMD in the lumbar spine vanished after adjustment for age, sex, weight and smoking history. In 3150 postmenopausal US women participating in NHANES, Wang et al. [26] found that the GNRI was positively associated with BMD at different femoral sites and inversely associated with osteoporosis. Similarly, in 7405 male and female NHANES participants aged 60 years or older, Huang et al. [24] identified an inverse association between GNRI and the presence of osteoporosis. The authors [24] therefore suggested that a low GNRI might be a risk factor for the development of osteoporosis. Our results are partly in contrast to the above. While we confirm a positive association of GNRI with heel stiffness index in underweight/normal weight individuals, a corresponding association was absent in the overweight/obese individuals, who constituted by far the largest part of our study population.

The discrepancy with the results of the previous studies from the general population [24,25,26] that identified associations across the entire population can be explained by differences in study population and methodology. For example, Qing et al. [25] examined predominantly underweight/normal weight individuals (mean BMI of 23.8 kg/m^2^ vs. 29.1 kg/m^2^ in SHIP). In the studies by Wang et al. and Huang et al. [24,26], the distribution of the GNRI is different, with much higher mean values and a wider range. Especially in the large study of Huang et al. [24], the distribution of the GNRI is hardly comparable to the one described here. The former describes GNRI scores ranging between 50 and 200, which seem questionable for data obtained from the general population. Moreover, Qing et al. [25] and Wang et al. [26] do not report whether they followed the recommendation of Bouillanne et al. [7] to set the actual weight/ideal weight to ‘1’ in individuals who exceeded their ideal weight in the calculation of the GNRI. This impacts the analyses and limits the comparability of the study results.

To the best of our knowledge, only one study [32] has examined the relation between the GNRI and BMD with a subgroup analysis stratified by BMI. That work [32] included 447 patients with type 2 diabetes mellitus. The authors found no association between the GNRI and total lumbar, total hip or femoral neck BMD in men with a BMI ≥ 24 kg/m^2^, while the respective associations were present in patients with lower BMI. These results align with ours. However, in female diabetes patients and in the overall study population, a strong association between the GNRI and BMD was observed in both BMI groups. The observation of a lack of association in individuals with higher BMI is therefore not novel and is not exclusive to our study. Once more, discrepancies in the characteristics of the study populations, namely, the markedly lower average BMI (24.4 kg/m^2^) observed in the diabetic patients [32] compared to our study participants and the slightly different categorization of BMI (≥24 vs. ≥25 kg/m^2^ in the present study), limit the comparability of the results. Furthermore, the authors did not declare whether the actual weight/ideal weight was set to ‘1’.

The relation between body anthropometry or composition and bone health is complex. On the one hand, underweight is a well-known major risk factor for osteoporosis [33]. It has consistently been demonstrated to contribute to low BMD and an increased fracture risk [33]. Overweight, on the other hand, is related to increased BMD due to its effects on mechanical loading and increased oestrogen availability (reviewed in [22]). Yet, an ever-increasing BMI is also related to an increased fracture risk (as reviewed in [34,35]). Thus, in a study including more than 12,000 elderly Finnish women, the highest hip fracture rates over a period of 25 years were observed in women with the lowest BMI and the second highest rates in women with the highest BMI, defined according to the fifth and ninety-fifth percentiles [36]. This suggests a U-shaped relation between BMI and fracture risk, which may aid in explaining our results. Thus, it can be speculated that while a higher GNRI is typically associated with a reduced fracture risk, this association is outweighed and interfered with by the adverse effects of a very high BMI on bone health. In addition, recent reviews and meta-analyses [23,35] report that obesity may confer an increased risk of fractures of the humerus, ankle, vertebrae, ribs and more, while it may be protective against hip, pelvis and wrist fractures. The complex effects of obesity on BMD and fracture risk are thought to be a combination of increased mechanical loading, a different pattern of falls, differences in intake of micronutrients such as calcium and vitamin D [37] and metabolic changes associated with the accumulation of adipose tissue. The latter includes effects of not only increased adipose-tissue-derived pro-inflammatory adipokines and cytokines as well as hormonal changes but also leptin resistance and bone marrow adiposity [22,35,38]. These effects as well as obesity-related conditions such as type 2 diabetes, metabolic syndrome and dyslipidaemia contribute to bone fragility despite increased bone mass [22,35,38]. As a result, they may outweigh the potential positive effects of a high GNRI on bone health and hence diminish its measurable association with fracture risk and stiffness index. Another factor interfering with the association of the GNRI and bone health parameters may be lean mass. A meta-analysis by Ho-Pham et al. [39] suggests that lean mass may exert a more pronounced effect on BMD than fat mass. This is potentially attributable to the adverse effects mentioned above. Given that the GNRI is a composite index that does not account for the balance of fat and lean mass, results regarding obese individuals may be less precise.

Since 83% of our study population was overweight or obese and the effects of the GNRI on the bone stiffness index were absent in this group, it is not surprising that no association was observed in the overall population. Furthermore, the small number of underweight/normal weight elderly individuals limited the statistical power of the analyses. Our results therefore need to be interpreted with caution. Nevertheless, the effects observed here are potentially clinically meaningful, especially as the association between the GNRI and stiffness index was independent of BMI within this group of individuals. Underweight individuals have a significantly lower BMD compared to normal weight individuals, particularly women [40]. Meta-analyses [41,42] demonstrated that a BMI of 20 kg/m^2^ is related to a hip fracture risk nearly twice as high as a BMI of 25 kg/m^2^ [41] and, likewise, that weight loss is associated with an increased risk of hip fracture (risk ratio of 1.84) [42]. Dramatically lower DXA T-scores at the hip and spine and high proportions of individuals with T-scores indicative of osteoporosis were further observed in anorectic vs. normal weight young premenopausal women [43]. An adequate supply of energy is essential for musculoskeletal health as bone remodelling is an energy-demanding process [44]. A lack of energy in underweight may thus contribute to an impairment of bone remodelling and BMD apart from contributing to muscle weakness and an increasing risk of falls [41]. Furthermore, it causes decreased growth hormone and oestrogen levels, which are protective for bone health [42]. In the case of involuntary weight loss following severe or chronic disease, the underlying disease further compromises bone health [42]. In addition to a relative lack of energy, an inadequately composed diet lacking calcium and protein, as well as the presence of insufficient levels of vitamin D, impairs bone health [1]. Calcium is the major mineral building bone matrix, while vitamin D stimulates calcium absorption and thus affects calcium and bone metabolism. Protein makes up about one-third of bone mass and is essential for bone growth and remodelling [45]. It also stimulates production of IGF-1 [46], which plays a crucial role in bone cell proliferation [46,47,48]. Protein also contributes to the preservation of muscle mass [49], which mechanistically and metabolically impacts bone tissue [50]. A higher protein intake has been demonstrated to be associated with a higher BMD and a slower rate of bone loss in the elderly, with some studies even arguing for protein intake above the currently recommended daily allowance [51]. As a marker of nutritional risk, the GNRI contains more information than simple anthropometric measures alone, which could help identify underweight/normal weight elderly men and women at increased fracture risk. These individuals might benefit from further evaluation of bone health and osteoporosis assessment. Moreover, it may support the clinician in selecting individuals for nutritional advice regarding bone health.

### Strengths and Limitations

The present study has its strengths and limitations. It shall be highlighted here that the participants from the SHIP cohorts were extensively examined and underwent a large range of standardized medical assessments. This allowed us to consider several covariates in our models. With 1417 individuals aged 65 to 90 years, our study population also covers a broad age range. Moreover, our study is the first to assess the relation between GNRI and QUS-based bone stiffness instead of using DXA. As previously stated, DXA measurements, which represent the gold standard for osteoporosis diagnosis and are included in the WHO definition of osteoporosis [3], were not performed in the SHIP cohorts for various reasons. This restricts the comparability between this study and previous studies that have determined BMD using DXA. Different technologies and measurement sites yield disparate information regarding bone properties. It is therefore not surprising that a recent study demonstrated low to modest correlations [52] between QUS and total body BMD. Nevertheless, QUS is an independent predictor of fracture [53] with power comparable to that of DXA [30]. Keeping in mind that fractures are the most important complication of osteoporosis, QUS represents a valuable tool for the assessment of bone health, particularly in research settings and in combination with clinical risk factors.

In addition to this, the following limitations apply to our study. The number of individuals with underweight/normal weight and the number of individuals with a low/moderate GNRI-related risk were both low. This might result from a certain self-selection of the participants, given that participation in a voluntary epidemiologic study requires physical and cognitive resources. Patients with severe osteoporosis and fracture history often suffer from pain and immobility, which may have led to refusal to participate in the study and contributed to their under-representation in the sample. The small sample size in the subgroup analysis limits its statistical power, which might have caused the borderline significant results in some of the analyses. Yet, the emergence of a significant result in the relatively small subgroup of underweight/normal weight individuals indicates that the study likely had sufficient power to detect an effect of the reported magnitude within this subgroup. To confirm the validity of our results, we suggest following up by attempting to replicate our findings in larger studies and including different geographic regions. Our study would also profit from the inclusion of data on bone turnover. Unfortunately, the measurement of bone turnover marker (BTM) concentrations was confined to a subset of the study population. Thus, only 103 of the 240 underweight/normal weight participants had BTM measurements. Due to this low number, we refrained from including those data in our analyses. We must further acknowledge that data on the physical activity of our participants were limited, preventing us from performing more detailed analyses than classifying the participants as physically inactive or not. Additionally, detailed data on dietary intake were missing; such data would have allowed a deeper insight into the examined relation. Finally, our cross-sectional study design does allow to determine whether the reported associations are causal.

## 5. Conclusions

In elderly men and women with underweight/normal weight from the general population, the GNRI is positively associated with the bone stiffness index and the related osteoporotic fracture risk. In this group, the GNRI may prove useful in identifying individuals with an elevated fracture risk. Yet, due to the low number of underweight/normal weight individuals, our results need validation in larger cohorts.

## Figures and Tables

**Table 1:** Characteristics of the study population.

Characteristics	GNRI—No Risk (n = 955)	GNRI—Low/Moderate Risk (n = 462)	*p*
Male, %	53.0	53.9	0.75
Age, years	71.4 (4.93)	72.8 (5.60)	<0.01
GNRI	101.7 (2.95)	94.8 (2.26)	<0.01
BMI, kg/m^2^	28.9 (4.16)	29.5 (5.03)	0.02
BMI categories, %			0.69
Normal weight	17.3	16.2	
Overweight	46.5	45.2	
Obese	36.2	38.5	
Physical inactivity, %	39.3	47.8	<0.01
Diabetes mellitus, %	21.8	31.8	<0.01
Liver disease, %	10.5	12.3	0.29
Menopausal hormone therapy in women, %	4.7	6.1	0.44
eGFR, mL/min/1.73 m^2^	77.2 (14.4)	74.9 (15.5)	<0.01
Stiffness index	87.0 (17.7)	87.2 (18.0)	0.85
QUS-based fracture risk, %			0.90
Low	49.0	48.9	
Moderate	39.7	39.0	
High	11.3	12.1	

Data are the mean (standard deviation) or proportions. Group differences were tested with *t*-tests (continuous data) or chi-squared tests (categorical data). BMI, body mass index; eGFR, estimated glomerular filtration rate; GNRI, Geriatric Nutritional Risk Index; QUS, quantitative ultrasound.

**Table 2:** Associations between the GNRI and the ultrasound-based stiffness index or the ultrasound-based osteoporotic fracture risk. Results from linear and logistic regression models were obtained in the total population and in subgroups according to BMI. All models were adjusted for sex, age, diabetes mellitus, physical inactivity, eGFR, liver disease, menopausal hormone therapy and BMI. In all models, an increase of one standard deviation (SD) in the GNRI (=4.24 points) was modelled. The following abbreviations are used herein: BMI, body mass index; CI, confidence interval; eGFR, estimated glomerular filtration rate; GNRI, Geriatric Nutritional Risk Index; OR, odds ratio; QUS, quantitative ultrasound; std err, standard error; β, β-coefficient.

**QUS-Based Stiffness Index—Linear Regression**				
**Exposure**	**Population**	**β**	**Std Err**	***p***
GNRI (per SD)	Total (n = 1417)	0.216	0.428	0.614
	Underweight/normal weight (n = 240)	2.688	1.000	0.007
	Overweight (n = 653)	−0.260	0.628	0.679
	Obese (n = 524)	−0.103	0.742	0.890
**QUS-Based Osteoporotic Fracture Risk (Low vs. Moderate/High Risk)—Logistic Regression**				
**Exposure**	**Population**	**OR**	**95% CI**	***p***
GNRI (per SD)	Total (n = 1417)	1.001	0.89–1.19	0.993
	Underweight/normal weight (n = 240)	1.423	1.04–1.94	0.026
	Overweight (n = 653)	0.911	0.77–1.08	0.269
	Obese (n = 524)	0.994	0.82–1.20	0.949

## Data Availability

Restrictions apply to the availability of data generated or analysed during this study to preserve patient confidentiality or because they were used under license. Data can be applied for by following a standardized procedure: http://www2.medizin.uni-greifswald.de/cm/fv/ship/daten-beantragen/ (accessed 11 December 2024).
